# Telerehabilitation for Stroke: A Personalized Multi-Domain Approach in a Pilot Study

**DOI:** 10.3390/jpm13121692

**Published:** 2023-12-06

**Authors:** Sara Federico, Luisa Cacciante, Roberto De Icco, Roberto Gatti, Johanna Jonsdottir, Chiara Pagliari, Marco Franceschini, Michela Goffredo, Matteo Cioeta, Rocco Salvatore Calabrò, Lorenza Maistrello, Andrea Turolla, Pawel Kiper, on behalf of RIN_TR_Group

**Affiliations:** 1 Laboratory of Healthcare Innovation Technology, IRCCS San Camillo Hospital, 30126 Venice, Italy; sara.federico@hsancamillo.it (S.F.); pawel.kiper@hsancamillo.it (P.K.); 2 Department of Brain and Behavioral Science, University of Pavia, 27100 Pavia, Italy; roberto.deicco@mondino.it; 3 Headache Science & Neurorehabilitation Center, IRCCS Mondino Foundation, 27100 Pavia, Italy; 4 Humanitas Clinical and Research Center, IRCCS, Rozzano, 20148 Milan, Italy; roberto.gatti@hunimed.eu; 5 Department of Biomedical Sciences, Humanitas University, Pieve Emanuele, 20148 Milan, Italy; 6 IRCCS Fondazione Don Carlo Gnocchi, 20148 Milan, Italy; jjonsdottir@dongnocchi.it (J.J.); cpagliari@dongnocchi.it (C.P.); 7 Department of Neurological and Rehabilitation Sciences, IRCCS San Raffaele Roma, 00163 Rome, Italy; marco.franceschini@sanraffaele.it (M.F.); michela.goffredo@sanraffaele.it (M.G.); matteo.cioeta@sanraffaele.it (M.C.); 8 Department of Human Sciences and Promotion of the Quality of Life, San Raffaele University, 00163 Rome, Italy; 9 IRCCS Centro Neurolesi “Bonino Pulejo”, 98121 Messina, Italy; roccos.calabro@irccsme.it; 10 IRCCS San Camillo Hospital, 30126 Venice, Italy; lorenza.maistrello@hsancamillo.it; 11 Department of Biomedical and Neuromotor Sciences—DIBINEM, Alma Mater Studiorum Università di Bologna, 40138 Bologna, Italy; andrea.turolla@unibo.it; 12 Unit of Occupational Medicine, IRCCS, Azienda Ospedaliero-Universitaria di Bologna, 40138 Bologna, Italy

**Keywords:** telerehabilitation, telemedicine, stroke, personalized medicine

## Abstract

Stroke, a leading cause of long-term disability worldwide, manifests as motor, speech language, and cognitive impairments, necessitating customized rehabilitation strategies. In this context, telerehabilitation (TR) strategies have emerged as promising solutions. In a multi-center longitudinal pilot study, we explored the effects of a multi-domain TR program, comprising physiotherapy, speech therapy, and neuropsychological treatments. In total, 84 stroke survivors (74 analyzed) received 20 tailored sessions per domain, addressing individual impairments and customized to their specific needs. Positive correlations were found between initial motor function, cognitive status, independence in activities of daily living (ADLs), and motor function improvement after TR. A lower initial health-related quality of life (HRQoL) perception hindered progress, but improved ADL independence and overall health status, and reduced depression correlated with a better QoL. Furthermore, post-treatment improvements were observed in the entire sample in terms of fine motor skills, upper-limb functionality, balance, independence, and cognitive impairment. This multi-modal approach shows promise in enhancing stroke rehabilitation and highlights the potential of TR in addressing the complex needs of stroke survivors through a comprehensive support and interdisciplinary collaboration, personalized for each individual’s needs.

## 1. Introduction

Stroke is a leading cause of long-term disability for approximately 5 million individuals worldwide, often resulting in motor, speech language and cognitive impairments that significantly affect patients’ health-related quality of life (HRQoL) [1]. Understanding the global impact of stroke and prioritizing comprehensive rehabilitation programs remains crucial. Tailored interventions, including multi-modal strategies and technology integration, hold promise for improving stroke care and HRQoL [2,3,4]. Telerehabilitation (TR) has emerged as a versatile alternative, allowing remote assessment and treatment through virtual reality (VR), addressing accessibility challenges. TR allows therapists to assess, treat, and monitor patients from a distance, eliminating geographical barriers and improving accessibility to care [5], thus offering a more flexible and accessible solution for post-stroke rehabilitation. The challenge becomes evident when addressing the intricate task of administering sufficient therapy to stroke survivors, who inherently require a multi-professional approach. In stroke survivors, the intensity of training, and not only its type, determines the long-term improvement in motor function [6,7]. This is also true in the field of language and communication disorders, where the most significant improvements are observed in patients with aphasia when they are subjected to high doses of treatment [8]. Furthermore, in addition to the treatment dosage, it is imperative to consider the relationship between clinical domains and their recovery patterns. Hybbinette et al. found a strong correlation between the severity of impairment in speech language and hand motor domains and the extent of recovery within the initial six months following a stroke [9]. Recovery ratios were significantly linked between hand motor function and speech language functions. This parallel recovery pattern suggests a shared plasticity mechanism driving recovery across the speech language and motor domains post-stroke. Similarly, Ginex et al. indicated that cognitive abilities beyond language skills could have an impact on motor rehabilitation outcomes [10]. The study emphasizes the significance of considering both linguistic and cognitive factors in stroke rehabilitation planning, particularly for patients with aphasia. Patients affected by stroke, therefore, have complex and interconnected needs, as multiple clinical domains are often involved, which can interact with each other. Addressing stroke as a multi-domain condition underscores the complex interplay of clinical aspects, requiring a holistic treatment approach. 

### 1.1. Rationale for the Study

VR plays a significant role in modern neurorehabilitation strategies. Its environment facilitates motor learning through complex scenarios, and real-time monitoring customizes interventions based on individual progress. VR can provide a multi-sensory environment that sustains engagement and enables more intense training [11]. VR can enhance motor learning and functional recovery; the combined sensory and motor training appears to be more beneficial compared to conventional methods focused solely on motor-oriented training, resulting in increased engagement in cognitive [4,12,13,14], social [15], and motor [3,16] activities of daily living (ADLs) and even potential neuronal repair in memory-related brain regions [12]. Resulting in good compliance [17], adherence, and satisfaction [18], VR also appears to have positive effects on reducing caregiver burden [19] and to be generally usable and acceptable with proper training and infrastructure [20]. Various VR systems can be used for TR application, which appears to be comparable to conventional in-person rehabilitation in improving motor function [21], especially for upper-extremity and balance recovery among stroke survivors in the chronic stage [18]. This occurs in various functional domains [22,23], also resulting in improvements in cognitive deficits [23], especially for executive function impairments [24], and in patients with post-stroke aphasia [25,26]. In this study, a model for integrated TR was devised with the aim of offering a holistic and patient-centered approach to stroke rehabilitation. The potential of this innovative approach lies in the ability to combine different strategies that have been shown to be more effective in stroke treatment, rather than focusing on a single clinical dimension at a time. This initiative could help to better understand the variables that impact individual patient responsiveness to TR [27]. Consequently, this could facilitate the tailoring of rehabilitation methodologies for individuals recovering from stroke. 

### 1.2. Objectives

In this paper, we investigated the clinical impact of the integrated multi-domain TR program on patients affected by stroke. We employed a range of measures to thoroughly evaluate the efficacy of this treatment and its potential impact on patient care. This study presents pilot findings exploring the effects of an integrated TR system targeting motor, language, and cognitive impairments concurrently in stroke survivors, tailored to individual deficits for personalized care and resource optimization. 

## 2. Materials and Methods

### 2.1. Study Design and Setting

The present study is part of a multi-center, longitudinal, pilot study aimed at examining the implementation of an integrated, multi-domain TR system. A total of 84 stroke survivors were recruited between March 2018 and December 2021 from 7 Italian Research Hospitals (IRCCS), namely: IRCCS San Camillo Hospital of Venice, IRCCS Fondazione Don Carlo Gnocchi Onlus of Milan, IRCCS Centro Neurolesi Bonino-Pulejo of Messina, IRCCS Mondino Foundation of Pavia, IRCCS San Raffaele Roma, IRCCS San Raffaele Pisana, and Istituto Clinico Humanitas of Milan, all part of the Italian Neuroscience and Rehabilitation Network. Out of these 84 patients, 7 dropped out due to internet connectivity issues during the study. Among the remaining 77 patients, 3 individuals who solely underwent speech language treatment (SLT) were excluded from the statistical analyses due to insufficient data. The study followed a pre/post-intervention design and was registered at clinicaltrials.gov (https://classic.clinicaltrials.gov/ct2/show/NCT05703906, accessed on 1 September 2023). It was conducted in accordance with the principles of the Helsinki Declaration and was approved by the Ethics Committee for Clinical Trials of the Province of Venice and IRCSS San Camillo (Coordinator Centre, Prot. 2017.16) and all other participant centers. Written and informed consent was obtained for all participants.

#### 2.1.1. Participants

Patients were recruited at the end of their hospitalization period to receive an integrated TR treatment. Outcome assessments were recorded at baseline and post-intervention to evaluate changes in motor, language and cognitive functions, independence in ADLs and HRQoL levels. Subjects were enrolled if they met the following inclusion criteria: (1) first ischemic stroke event documented by CT/MRI; (2) time elapsed from stroke event ranged from 2 to 18 months; (3) age ranged from 18 to 80 years; (4) signed informed consent for participation in the study. Patients were excluded if they had (1) presence or history of cognitive decline (i.e., Montreal cognitive assessment score < 17.54), (2) recent fractures, (3) history of psychiatric disorders, or (4) presence or history of other neurological and/or internal diseases that could have interfered with the outcomes. 

#### 2.1.2. Intervention

The intervention consisted of a 20-session synchronous TR treatment for motor, cognitive, and/or language domains, 1 h/session, 5 times a week, for 4 weeks. This personalized TR approach allowed patients to engage in sessions tailored to their specific impairments, whether for a single affected domain, a combination of two domains, or all three. Patients performed exercises at home using a dedicated VRRS (Virtual Reality Rehabilitation System) home tablet kit (Khymeia Group, Noventa Padovana, Italy), with remote therapist supervision. The integrated TR system combined video conferencing with specialized software (VRRS) and tools for motor, cognitive, and language rehabilitation, facilitating real-time interaction between therapists and participants in personalized sessions. The system provided automatic quantitative and qualitative feedback, supplemented by continuous professional feedback for each module, and exercise customization based on individual abilities and goals. For motor TR, inertial sensors (Khymu) and a grasping detection sensor (K-wand) (Khymeia Group, Noventa Padovana, Italy) were utilized to interact with VR exercises on the provided tablet, focusing on upper-limb rehabilitation and reaching and catching movements. In particular, participants had to perform a reaching or a grasping movement within a VR environment by using a motion detection sensor that, through a light sensor, can be recognized by the VRRS system so that it can track the trajectories and movements performed by the patients. Some exercises used a gamification system, with movements to be performed to control an effector that could consisted, for example, of a spacecraft or a balloon, whereas other exercises provided visual cues for the reaching of a dot. In both cases, visual and auditory feedback was provided by the system to correct the movement. The difficulty of the exercises could be changed by modifying the time and the number of repetitions for completing the task, together with changing the sensitivity of the devices. Cognitive and SLT exercises were conducted using the tablet’s touch screen, targeting attention, memory, problem-solving, and executive functions remotely. Cognitive training used exercises targeting especially attentive functions, by using exercises based on attentive matrices in which participants had to find a target stimulus within a matrix of distractors. SLT-TR was delivered through video conference to address lexical retrieval impairment. The exercises were based on naming training, with the word target delivered through visual, written, or auditory channels. The difficulty of the exercises could be managed by modulating the word length, frequency of use, time, and number of repetitions given to patients to complete the task. Patients received a suitcase containing the tablet and devices at their home, where they connected the tablet to their home internet. During the final week of their hospital stay, patients received training on the device, and technical assistance was readily available throughout the treatment period. 

#### 2.1.3. Outcome Assessment

Evaluations and clinical data collection were performed by a trained physiotherapist, speech language therapist, or neuropsychologist in relation to the domain that needed to be assessed at the beginning of the treatment program (T0) and at the end of 20 sessions of TR (T1).

##### Change in Motor Function

- The Fugl-Meyer assessment—upper extremity (FMA-UE) is a stroke-specific scale assessing the motor functioning with scores. There are 3 values for each of the 5 domains: 0 (severe impairment), 1 (moderate impairment), 2 (preserved function). The 5 domains assessed include motor function (upper-limb maximum score = 66; lower-limb maximum score = 34), sensory function (maximum score = 24), balance (maximum score = 14), joint range of motion (maximum score = 44), and joint pain (maximum score = 44), for an overall maximum score of 226 points [28].
- The nine-hole pegboard test (NHPT) measures dexterity and fine motor coordination by evaluating the timing of peg insertion and removal speed [29].

##### Change in Linguistic Function

- The Aachener aphasia test (AAT) is an impairment-centered aphasia test battery known for its good construct validity and test–retest reliability, focused on five subscales: token test, repetition, naming, writing, and comprehension [30].

##### Change in Cognitive Function

- The Montreal cognitive assessment (MoCA) screens cognitive function across various domains, including visuospatial/executive skills, naming, memory, attention, comprehension, abstraction, delayed recall, and orientation. The total possible score is 30, corresponding to a good performance [31].

##### Change in Independence (Activities of Daily Living, ADLs)

- The Barthel index (BI) assesses 10 ADLs and mobility, with scores ranging from 0 (dependent) to 10 (independent) [32].

##### Change in HRQoL Levels

- The Short Form 36 (SF-36) questionnaire consists of 36 questions that are divided into physical functioning, role limitations due to physical health problems, bodily pain, general health perceptions, vitality, social functioning, role limitations due to emotional problems, and mental health categories. It is scored from 0 to 100, with higher scores indicating a better HRQoL [33].

##### Change in Behavior (Depression)

- The Beck depression inventory (BDI) is a self-reported questionnaire designed to assess the presence and severity of depressive symptoms in individuals. The total score ranges from 0 to 63, with scores > 10 indicative of depressive symptomatology [34].

### 2.2. Statistical Analysis

The main demographics and clinical characteristics of the sample were analyzed by calculating the mean, standard deviation, frequency, and percentage values. To determine the presence of a statistically significant difference in the various clinical domains after TR, values of pre-treatment (T0) variables were compared with post-treatment (T1) variables using a Student’s *t*-test for paired data or the Wilcoxon test, depending on the distribution of data, which was investigated by means of the Shapiro–Wilk test. To assess potential differences in therapy effectiveness based on the type of rehabilitation administered, variations from pre- to post-therapy between therapy groups were compared. This comparison was executed using a one-way ANOVA or Kruskal–Wallis test. In the event of significant differences between the groups, post hoc tests were conducted using Holm’s correction. Generalized linear regression models (GLRMs) were estimated to infer any potential causal relationship between the motor impairment index (i.e., FMA-UE-Motor), set as the dependent variable, and post-treatment clinical and demographic outcomes (i.e., sex, age, time from event, BDI, BI, MoCA, SF-36, GH, number of treatment sessions, and type of treatment), set as the independent variables. Exploratory GLRMs were also conducted to investigate potential causal relationships between the HRQoL level (measured through SF-36-GH) as the dependent variable, and various post-treatment clinical and demographic outcomes, including sex, age, time from event, BDI, BI, MoCA, number of treatment sessions, type of treatment, and FMA-UE-Motor, as the independent variables. Each regression model’s fitting was evaluated using McFadden’s index of explained variance (pseudo-R^2^) and the model residual analysis [35,36]. The statistical significance level was set at *p* < 0.05 and all the statistical analyses were performed using the free software R Studio 4.3.1 [37].

## 3. Results

### 3.1. Sample Characteristics

Demographic characteristics are summarized in Table 1 for the whole sample (n = 74 patients) and the samples divided by type of treatment received (Table 2). The average age of the patients was 61 (±12) years; 50 (68%) were male and 24 (32%) were female. The time from stroke onset was 4.9 (±3.4) months; 32 patients (43%) had lesions on the right hemisphere and 42 patients (57%) on the left side. They had received 10 (±3) years of schooling and the mean number of therapy sessions attended by the patients was 19 (±6). In the study, patients received treatment tailored to their specific needs. Consequently, 23 patients underwent only motor TR (M-TR), 40 received a combination of motor and cognitive TR (M + C-TR), and 11 underwent the comprehensive treatment, which included motor, cognitive, and SLT-TR (M + C + SLT-TR).

### 3.2. Effectiveness of Therapy across the Entire Sample

The results of the overall sample test demonstrated improvements in several key areas (Table 3). Notably, improvements were observed in fine motor skills (NHPT: V = 107; *p* < 0.001), upper-limb functionality (FMA-UE-Motor: V = 280.5; *p* < 0.001; FMA-UE-Pain-ROM: V = 364; *p* < 0.001) and balance (FMA-Balance: V = 188.5; *p* = 0.002). Additionally, there were improvements in terms of independence (BI: V = 161.5; *p* < 0.001) and the participants’ HRQoL, particularly in relation to physical functioning (SF-36-PF: V = 277; *p* = 0.029). Furthermore, cognitive impairment also showed improvement (MoCA: V = 247.5; *p* < 0.001).

### 3.3. Comparison of Different Types of Rehabilitation Performed

Table 4 shows the results of tests conducted to compare changes in outcome values among the three types of therapy received. The M-TR group showed improvements in mobility, joint flexibility, and pain reduction, as well as enhancements in balance, independence, and cognitive function. Similar improvements were observed in the group that received both motor and cognitive treatments (M + C-TR), with an additional increase in the HRQoL concerning physical and social functioning. Meanwhile, the group that received comprehensive treatment reported changes in fine motor skills. Only two of the analyzed variables exhibited significant changes among the three groups of patients. Specifically, differences were observed for the variable NHPT (Chi-sq = 10.91; *p* = 0.004) and the variable SF-36-PF (Chi-sq = 6.86; *p* = 0.032). Post hoc analyses (Appendix A, Table A1) indicated that the improvement was more pronounced in patients who received motor and cognitive therapy as well as all three types of therapy when compared to those who received only motor therapy.

### 3.4. General Linear Regression Models

In the first GLRM (Table 5), the post-treatment Fugl-Meyer assessment—upper extremity, the motor subcomponent (FMA-UE-Motor) outcome was significantly associated with its pre-treatment value ($\hat{\beta }$ = 0.79, *p* < 0.001), MoCA ($\hat{\beta }$ = 0.44, *p* = 0.003), BI ($\hat{\beta }$ = 0.22, *p* = 0.011), and SF-36-GH ($\hat{\beta }$ = −0.16, *p* = 0.007). The coefficient of determination of the model was pseudo-R^2^ = 0.90 and the model residuals were normally distributed (*p* = 0.224). 

As for the model relative to the SF-36-GH outcome (Table A2), there was a significant relation among Tot BI ($\hat{\beta }$ = 0.26; *p* = 0.008) and SF-36-GH—baseline ($\hat{\beta }$ = 0.89, *p* < 0.001). The coefficient of determination of the model was pseudo-R^2^ = 0.56 and the residuals of the model were normally distributed (*p* = 0.764). However, it is worth noting that some variables, though not significant, would contribute to better explaining the variance of the model, potentially increasing the index from 0.56 to 0.59 (Table 6). Specifically, these variables are months since injury ($\hat{\beta }$ = −0.62, *p* = 0.147) and Tot BDI ($\hat{\beta }$ = 0.313; *p* = 0.158). Once again, the model residuals followed a normal distribution (*p* = 0.620).

## 4. Discussion

The implementation of this personalized and multi-domain TR for stroke survivors led to varying degrees of improvement among different treatment groups. The study found improvements across various areas in the entire sample, including fine motor skills, upper-limb functionality, balance, independence, cognitive impairment, and HRQoL. When comparing different rehabilitation types, each group showed distinct but meaningful improvements. The M-TR group demonstrated enhancements in mobility, joint flexibility, pain reduction, balance, independence, and cognitive function. Similarly, the M + C-TR group exhibited pronounced improvements, with additional enhancements in the HRQoL domains related to physical and social functioning. Meanwhile, the comprehensive treatment group showed notable changes, specifically in fine motor skills. Differences in therapeutic outcomes among the groups may be linked to variations in neurophysiological recovery potential due to the diverse stages of stroke recovery at baseline. Indeed, in our sample, the group receiving solely motor therapy was in the chronic stage, while the other two groups were in the late subacute stage. It is well known that, while behavioral changes can manifest years after a stroke, the spontaneous neurological recovery follows a logistic pattern that plateaus within the first 10 weeks after the incident [11,38,39]. Challenges faced by patients in the chronic stage of stroke recovery differ from those in the subacute stage, encompassing potential limitations in neuroplasticity and a comparatively slower rate of functional improvement.

Our analysis explored the factors contributing to the motor function (FMA-UE-Motor) and HRQoL (SF-36-GH) improvements achieved with TR for stroke survivors. Through linear regression analysis, we found that motor function positively correlated with baseline motor function, cognitive status, and independence in daily life, but negatively correlated with baseline HRQoL perception, with a very strong correlation (R^2^ = 0.90) (Table 5). This analysis highlights that the initial level of motor function, cognitive status, and the patient’s independence in ADLs are crucial factors influencing the improvement of motor function during TR. Additionally, it suggests that a lower baseline perception of overall HRQoL may present obstacles to achieving substantial improvements in motor function through TR. These findings underscore the importance of considering these variables when designing tailored TR interventions to meet the specific needs of stroke survivors.

The specific correlation analysis for the perceived general HRQoL in stroke survivors was positively correlated (R^2^ = 0.59) with their independence in ADLs, their baseline general health status, and the level of patient depression. Conversely, a negative correlation was found between perceived HRQoL and the time elapsed since the stroke occurred. This correlation can be considered moderately strong (Table 6). These findings suggest that the implementation of TR programs aimed at enhancing independence in ADLs, optimizing overall health management, and providing effective support for mental health care may have a significant impact on the overall rehabilitation experience and HRQoL for stroke survivors. Considering the potential of TR in offering continuous and personalized remote support, an integrated approach that addresses both physical and cognitive rehabilitation aspects could lead to lasting improvements in functionality and overall well-being for these patients, thus contributing to a better long-term HRQoL. As per the existing literature, a virtual environment can amplify social interactions [15], engagement in rehabilitation processes, attention, memory, and overall well-being [4]. This effect becomes even more pronounced when these activities are conducted at home through TR. The overall clinical improvement in stroke survivors undergoing multi-domain TR can be linked to the stimulation of multiple sensory components [40], facilitated by VR, along with its capacity to offer diverse rehabilitation modalities, potentially identifying analogous recovery patterns [9,10]. Our results did not yield any evidence of a relationship between the therapy dose and clinical improvement, including various therapy combinations, despite the demonstrated efficacy of different therapy types.

Our study introduces an integrated TR system that facilitates a comprehensive and holistic approach to address diverse aspects of stroke recovery, encompassing motor, cognitive, and language rehabilitation services. This innovation presents two notable advantages. Firstly, it offers a convenient solution for individuals constrained by geographical limitations, reduced mobility, or time constraints, enabling remote access to rehabilitation services. Secondly, it promotes interdisciplinary collaboration among therapists with varying specialties, thereby ensuring cohesive and comprehensive patient care. By providing high-quality rehabilitation services at home, the system enhances accessibility and sustains continuous care. The system’s ability to target multiple dimensions of rehabilitation, spanning from motor skills, cognitive function, and language abilities to independence in ADLs, highlights its potential as an impactful tool in stroke rehabilitation, particularly beneficial in regions with limited resources and inadequate neuro-rehabilitation expertise and facilities [23]. The observed improvements not only contribute to the overall well-being of patients but also suggest promising implications for the broader application of TR in neurological care. Our study advocates for a multi-modal approach in TR, emphasizing the potential for improved outcomes in stroke rehabilitation. This study represents an exploratory pilot investigation and further research is essential to validate these findings. Long-term effects and a more comprehensive understanding of the impact on HRQoL require further investigation [18,41]. Employing mixed-methods approaches to understand contextual factors influencing care and rehabilitation through TR can refine the personalization of therapy. Moreover, extensive investigations are necessary to predict the patient characteristics that are conducive to receiving and benefiting from TR. 

### Limitations

This study is a pilot study with a single experimental group. Therefore, the absence of a control group poses a challenge in establishing the intervention’s effectiveness as it becomes difficult to differentiate the impact of the intervention from external variables or potential confounding factors. Another limitation arises from the lack of long-term follow-up, preventing the assessment of the intervention’s enduring effects.

## 5. Conclusions

The findings of this pilot study suggest potential benefits of the integrated, multi-domain, and personalized TR system for stroke survivors, indicating promising improvements across various domains. The observed associations underscore the potential of comprehensive TR support in mitigating post-stroke challenges and enhancing patients’ HRQoL. Furthermore, the results emphasize the importance of considering psychological well-being within TR programs, highlighting its potential contribution to the holistic recovery of stroke survivors.

## Figures and Tables

**Table 1:** Demographic and clinical characteristics of the patients.

Characteristic	Patients (n = 74)
Sex, n (%)	
Female	24 (32%)
Male	50 (68%)
Age, mean (SD)	61 (±12)
Education, mean (SD)	10 (±3)
Lesioned hemisphere n (%)	
right	32 (43%)
left	42 (57%)
Time from stroke, mo., mean (SD)	4.9 (±3.4)
No. of sessions, mean (SD)	19 (±6)
Type of TR treatment, n (%)	
M-TR	23 (31%)
M + C-TR	40 (54%)
M + C + SLT-TR	11 (15%)

Values are expressed as mean ± standard deviation (SD) for quantitative measures, and as absolute frequencies (n) and percentages (%) for discrete variables. Abbreviations: TR = telerehabilitation; M-TR = motor telerehabilitation; M + C-TR = motor and cognitive telerehabilitation; M + C + SLT-TR = motor, cognitive, and speech therapy telerehabilitation.

**Table 2:** Demographic and clinical characteristics of the patients, stratified by type of treatment.

Characteristic	M-TR n = 23	M + C-TR n = 40	M + C + SLT-TR n = 11	*p*-Value
Sex, n (%)				*p* = 0.003 *
Female	14 (61%)	8 (20%)	2 (18%)	
Male	9 (39%)	32 (80%)	9 (82%)	
Age, mean (SD)	64 (±11)	57 (±12)	67 (±10)	*p* = 0.024 *
Education, mean (SD)	10 (±3)	11 (±4)	10 (±3)	*p* = 0.7
Lesioned Hemisphere, n (%)				*p* = 0.4
right	8 (35%)	20 (50%)	4 (36%)	
left	15 (65%)	20 (50%)	7 (64%)	
Time from stroke, mo., mean (SD)	7.0 (±3.3)	3.7 (±2.9)	3.8 (±2.3)	*p* < 0.001 *
No. of sessions, mean (SD)	16 (±6)	21 (±3)	19 (±10)	*p* = 0.005 *

Fisher’s exact test; Kruskal–Wallis rank sum test. Values are expressed as mean ± standard deviation (SD) for quantitative measures, and as absolute frequencies (n) and percentages and (%) for discrete variables; * *p*-value statistically significant < 0.05. Abbreviations: TR = telerehabilitation; M-TR = motor telerehabilitation; M + C-TR = motor and cognitive telerehabilitation; M + C + SLT-TR = motor, cognitive, and speech therapy telerehabilitation.

**Table 3:** Effectiveness of therapy in whole sample.

Outcomes	T0		T1		*p*-Value
	Mean	(SD)	Mean	(SD)	
NHPT	0.29	0.20	0.36	0.24	*p* < 0.001 *
FMA-UE-Motor	48.69	16.02	52.13	14.83	*p* < 0.001 *
FMA-UE-Pain-ROM	40.53	7.42	42.82	5.34	*p* < 0.001 *
FMA-UE-Sensitivity	20.12	5.75	20.31	5.23	*p* = 0.074
FMA-Balance	11.54	2.58	12.06	2.07	*p* = 0.002 *
BI	82.40	25.07	86.58	21.35	*p* < 0.001 *
MoCA	22.19	5.28	23.44	4.48	*p* < 0.001 *
BDI	8.22	7.36	7.77	7.06	*p* = 0.229
SF-36-PF	54.96	24.21	58.36	24.68	*p* = 0.029 *
SF-36-RP	58.60	27.06	61.29	28.41	*p* = 0.353
SF-36-RE	69.90	31.68	73.24	31.58	*p* = 0.191
SF-36-VT	56.35	22.12	55.29	22.35	*p* = 0.506
SF-36-MH	59.22	22.89	60.33	23.07	*p* = 0.411
SF-36-SF	52.21	22.05	50.45	20.19	*p* = 0.316
SF-36-BP	32.40	20.28	31.82	20.48	*p* = 0.574
SF-36-GH	67.48	10.00	58.18	23.49	*p* = 0.136

Values are expressed as means (SD). * *p* < 0.05; Wilcoxon sign rank test. Abbreviations: T0 = pre-therapy variable; T1 = post-therapy variable; NHPT = nine-hole pegboard test: results from affected side, expressed in terms of pegs-to-time ratio. FMA-UE = Fugl-Meyer assessment–upper extremity; subcomponents: FMA-UE-Motor = motor function; FMA-UE-Pain-ROM = joint pain and range of motion; FMA-UE-Sensitivity = sensory functioning. BI= Barthel index; MoCA = Montreal cognitive assessment; BDI = Beck’s depression inventory. SF-36 = Short Form 36; subcomponents: PF = physical functioning; RP = role—physical; RE = role—emotional; VT = vitality; MH = mental health; SF = social functioning; BP = bodily pain; GH = general health.

**Table 4:** Effectiveness of therapy in each treatment type.

Outcomes	M-TR (n = 23)			M + C-TR (n = 40)			M + C + SLT-TR (n = 11)		
	T0	T1		T0	T1		T0	T1	
	Mean (SD)	Mean (SD)	*p*-Value	Mean (SD)	Mean (SD)	*p*-Value	Mean (SD)	Mean (SD)	*p*-Value
NHPT	0.31 (0.20)	0.32 (0.20)	*p* = 0.266	0.28 (0.22)	0.37 (0.27)	*p* < 0.001 *	0.30 (0.16)	0.40 (0.24)	*p* = 0.004 *
FMA-UE-Motor	41.30 (16.80)	44.91 (14.48)	*p* = 0.021 *	49.40 (15.19)	53.31 (15.09)	*p* < 0.001 *	61.55 (6.68)	62.36 (4.48)	*p* = 0.589
FMA-UE-Pain-ROM	36.57 (7.12)	39.30 (5.56)	*p* = 0.022 *	41.35 (7.20)	43.97 (4.44)	*p* = 0.005 *	45.82 (4.35)	46.09 (4.11)	*p* = 1
FMA-UE Sensitivity	18.22 (6.62)	18.55 (5.30)	*p* = 0.293	20.25 (5.58)	20.41 (5.49)	*p* = 0.083	23.64 (1.21)	23.45 (1.81)	*p* = 1
FMA-Balance	10.65 (2.29)	11.82 (2.42)	*p* < 0.001 *	12.21 (2.76)	12.19 (1.89)	*p* = 0.681	11.36 (2.16)	12.18 (1.94)	*p* = 0.058
BI	80.04 (19.26)	82.74 (18.48)	*p* = 0.021 *	80.31 (30.11)	85.87 (24.91)	*p* < 0.001 *	94.73 (8.16)	97.09 (3.91)	*p* = 0.147
MoCA	21.50 (6.72)	23.17 (4.92)	*p* = 0.008 *	21.81 (4.67)	23.22 (4.25)	*p* = 0.005 *	24.81 (3.89)	24.64 (4.60)	*p* = 0.799
BDI	11.67 (7.93)	10.39 (7.66)	*p* = 0.337	7.56 (7.23)	7.43 (6.95)	*p* = 0.638	4.91 (4.70)	4.64 (5.22)	*p* = 0.404
SF-36-PF	58.50 (16.29)	54.44 (17.17)	*p* = 0.262	51.15 (27.31)	56.29 (29.00)	*p* = 0.009 *	63.80 (21.34)	73.30 (9.44)	*p* = 0.093
SF-36-RP	64.06 (20.12)	67.56 (15.07)	*p* = 0.635	51.40 (28.35)	54.03 (32.04)	*p* = 0.362	77.60 (22.66)	77.60 (24.20)	*p* = 0.916
SF-36-RE	71.39 (21.89)	76.89 (16.19)	*p* = 0.330	65.88 (36.38)	68.42 (38.11)	*p* = 0.456	83.30 (23.57)	85.00 (21.42)	*p* = 1
SF-36-VT	53.94 (10.26)	52.89 (11.70)	*p* = 0.507	54.75 (27.34)	53.29 (27.59)	*p* = 0.601	67.10 (7.32)	67.20 (5.83)	*p* = 1
SF-36-MH	57.39 (11.83)	58.89 (10.53)	*p* = 0.979	58.53 (28.49)	59.18 (29.34)	*p* = 0.475	65.30 (8.31)	67.30 (5.68)	*p* = 0.475
SF-36-SF	50.56 (13.05)	54.44 (12.47)	*p* = 0.497	52.50 (27.06)	47.89 (24.51)	*p* = 0.044 *	54.00 (10.75)	53.00 (10.59)	*p* = 0.850
SF-36-BP	38.78 (14.77)	39.17 (15.75)	*p* = 0.697	30.15 (21.95)	27.87 (21.47)	*p* = 0.412	29.9 (21.21)	33.6 (22.11)	*p* = 0.462
SF-36-GH	64.22 (10.40)	58.67 (11.80)	*p* = 0.111	69.09 (10.16)	55.68 (29.40)	*p* = 0.794	68.00 (8.00)	66.80 (7.79)	*p* = 0.587

Values are expressed as means (SD). * *p* < 0.05; Wilcoxon sign rank test. Abbreviations: n = number of patients; T0 = pre-therapy variable; T1 = post-therapy variable; TR = telerehabilitation; M-TR = motor telerehabilitation; M + C-TR = motor and cognitive telerehabilitation; M + C + SLT-TR = motor, cognitive, and speech therapy telerehabilitation. NHPT = nine-hole pegboard test: results from affected side, expressed in terms of pegs-to-time ratio. FMA-UE = Fugl-Meyer assessment—upper extremity; subcomponents: FMA-UE-Motor = motor function; FMA-UE-Pain-ROM = joint pain and range of motion; FMA-UE-Sensitivity = sensory functioning. BI = Barthel index; MoCA = Montreal cognitive assessment; BDI = Beck’s depression inventory. SF-36 = Short Form 36; subcomponents: PF = physical functioning; RP = role—physical; RE = role—emotional; VT = vitality; MH = mental health; SF = social functioning; BP = bodily pain; GH = general health.

**Table 5:** Relationship between FMA-UE-Motor and clinical scales.

Regression Model	β ± SE	Pseudo-R^2^	*p*-Value_res_
**FMA-UE-Motor—Post-Treatment**		0.90	*p* = 0.224
Intercept	−7.51 ± 6.3		
FMA-UE-Motor—Baseline	0.79 ± 0.06		
MoCA	0.44 ± 0.17		
BI	0.22 ± 0.07		
SF-36-GH—Baseline	−0.16 ± 0.06		

The outcomes are displayed with the estimate of regression coefficient with standard error (β ± SE), McFadden’s index of explained variance (pseudo-R^2^), and the *p*-value of the test for the normal distribution of residuals (*p*-value_res_).

**Table 6:** Relationship between SF-36 general health and clinical scales.

Regression Model	β ± SE	Pseudo-R^2^	*p*-Value_res_
**SF-36-GH—Post-Treatment**		0.59	*p* = 0.620
Intercept	−18.42 ± 14.53		
BI	0.25 ± 0.11		
SF-36-GH—Baseline	0.91 ± 0.13		
Time from stroke, mo.	−0.62 ± 0.42		
BDI	0.31 ± 0.22		

The outcomes are displayed with the estimate of regression coefficient with standard error (β ± SE), McFadden’s index of explained variance (pseudo-R^2^), and the *p*-value of the test for the normal distribution of residuals (*p*-value_res_).

## Data Availability

The datasets that support the findings of this study are available from the corresponding author upon request.

## Appendix A

**Table A1:** Difference in outcomes values among the three therapy groups.

Outcomes	M-TR (n = 23)		M + C-TR (n = 40)		M + C + S-TR (n = 11)		*p*-Value
	**Mean**	**SD**	**Mean**	**SD**	**Mean**	**SD**	
NHPT	0.01	0.05	0.09	0.12	0.13	0.18	*p* = 0.004 *
FMA-UE-Motor	1.91	7.02	4.03	5.65	0.82	3.46	*p* = 0.168
FMA-UE-Pain-ROM	2.74	5.22	2.62	6.07	0.27	2.20	*p* = 0.276
FMA-UE Sensitivity	0.59	3.51	0.26	2.81	-0.18	2.27	*p* = 0.444
FMA-UE-Balance	1.14	1.32	−0.09	2.18	0.82	1.17	*p* = 0.059
BI	2.70	5.28	5.56	9.99	2.36	5.77	*p* = 0.764
MoCA	1.67	2.58	1.62	3.19	−0.16	2.50	*p* = 0.289
BDI	−0.82	7.24	−0.08	6.12	−0.27	6.40	*p* = 0.838
SF-36-PF	−4.06	11.84	5.61	12.27	9.50	14.59	*p* = 0.032 *
SF-36-RP	3.50	18.23	2.89	19.79	0.00	28.92	*p* = 0.837
SF-36-RE	0.01	0.05	0.09	0.12	0.13	0.18	*p* = 0.777
SF-36-VT	1.91	7.02	4.03	5.65	0.82	3.46	*p* = 0.561
SF-36-MH	2.74	5.22	2.62	6.07	0.27	2.20	*p* = 0.723
SF-36-SF	0.59	3.51	0.26	2.81	−0.18	2.27	*p* = 0.238
SF-36-BP	1.14	1.32	−0.09	2.18	0.82	1.17	*p* = 0.575
SF-36-GH	2.70	5.28	5.56	9.99	2.36	5.77	*p* = 0.509

Values are expressed as means (SD). * *p* < 0.05; Kruskal–Wallis rank sum test. Abbreviations: n = number of patients; TR = telerehabilitation; M-TR = motor telerehabilitation; M + C-TR = motor and cognitive telerehabilitation; M + C + SLT-TR = motor, cognitive, and speech therapy telerehabilitation. NHPT = nine-hole pegboard test: results from affected side, expressed in terms of pegs-to-time ratio. FMA-UE = Fugl-Meyer assessment–upper extremity; subcomponents: FMA-UE-Motor = motor function; FMA-UE-Pain-ROM = joint pain and range of motion; FMA-UE-Sensitivity = sensory functioning. BI = Barthel index; MoCA = Montreal cognitive assessment; BDI = Beck’s depression inventory. SF-36 = Short Form 36; subcomponents: PF = physical functioning; RP = role—physical; RE = role—emotional; VT = vitality; MH = mental health; SF = social functioning; BP = bodily pain; GH = general health.

**Table A2:** Relationship between SF-36 general health and clinical scales.

Regression Model	β ± SE	Pseudo-R^2^	*p*-Value_res_
**SF-36-GH—Post Treatment**		0.56	*p* = 0.764
Intercept	−18.42 ± 11.72		
BI	0.26 ± 0.09		
SF-36-GH—Baseline	0.89 ± 0.13		

The outcomes are displayed with the estimate of regression coefficient with standard error (β ± SE), McFadden’s index of explained variance (pseudo-R^2^), and the *p*-value of the test for the normal distribution of residuals (*p*-value_res_).
